# Combining statistics: the role of phonotactics on cross-situational word learning

**DOI:** 10.1186/s41155-022-00234-y

**Published:** 2022-09-28

**Authors:** Rodrigo Dal Ben, Débora de Hollanda Souza, Jessica F. Hay

**Affiliations:** 1 Department of Psychology, Universidade Federal de São Carlos, São Carlos, SP Brazil; 2 Department of Psychology, University of Tennessee, Knoxville, TN USA

**Keywords:** Statistical learning, Phonotactic probability, Cross-situational word learning, Language learning, Word learning

## Abstract

Language learners can rely on phonological and semantic information to learn novel words. Using a cross-situational word learning paradigm, we explored the role of phonotactic probabilities on word learning in ambiguous contexts. Brazilian-Portuguese speaking adults (*N* = 30) were exposed to two sets of word-object pairs. Words from one set of labels had slightly higher phonotactic probabilities than words from the other set. By tracking co-occurrences of words and objects, participants were able to learn word-object mappings similarly across both sets. Our findings contrast with studies showing a facilitative effect of phonotactic probability on word learning in non-ambiguous contexts.

## Introduction

Most everyday word learning unfolds in phonologically rich and referentially ambiguous contexts (Quine, 1960). One phonological regularity that has been shown to influence word learning is phonotactic probability, which can be defined as positional statistics that represent how frequently phonological segments happen together in a given language (Vitevitch & Luce, 2004). Words with higher phonotactic probabilities contain common segments from a language, whereas words with lower phonotactic probabilities contain less frequent segments. For instance, due to variations in the phonotactic probabilities of their initial biphones, the word *fall* has an overall higher phonotactic probability than the word *tall* but has a lower phonotactic probability than the word *call*.^1^ One way to investigate the role phonotactic probabilities play in word learning is to create novel words with different degrees of phonotactic probabilities and then pair them with novel referents. By doing so, research has shown that, across the lifespan, words with higher phonotactic probabilities are learned faster and with greater accuracy than words with lower phonotactic probabilities (e.g., Benitez & Saffran, 2021; Estes & Bowen, 2013; Estes et al., 2011; Steber & Rossi, 2020; Storkel et al., 2013; Sundara et al., 2022; but see Cristia, 2018). Most of these studies use unambiguous word learning paradigms, in which one novel word is paired with one novel referent in a given trial. In natural contexts, however, word learning usually unfolds across ambiguous contexts (e.g., Clerkin et al., 2017). Thus, it is important to understand how phonotactic probabilities impact word learning in these more ecologically relevant (i.e., ambiguous) contexts.

Work by Fitneva et al. (2009) has begun to shed some light on this issue by investigating how a related phonological cue, namely phonological typicality, impacts word learning in ambiguous contexts. Phonological typicality indexes how typical the phonology of a word is in relation to its lexical category (e.g., verbs, nouns, adjectives). It is calculated by computing the distance of each phonemes’ features, in each word position, in relation to words from the same lexical category. Children (7-years-old) were presented with ambiguous trials with a novel word and two pictures depicting actions or objects. Novel words had different degrees of phonological typicality as verbs or nouns. Results showed that participants relied on typicality to make initial associations, choosing actions or objects as a function of the labels’ typicality as verbs or nouns. Following their initial associations, they received feedback on their choices. Once feedback started, participants ignored typicality and relied on mutual exclusivity to map novel words. The authors argue that by relying on phonological information to make initial guesses, learners would be better situated to learn word-referent relations in complex ambiguous contexts, and this initial bias could have a cascading effect on word learning.

One semantic regularity that does not rely on contingent feedback and that can help solve referential ambiguity is the co-occurrence of words and referents. For instance, in a seminal study, Yu and Smith (2007) showed that by comparing word-referent co-occurrences across ambiguous trials, adults were able to solve the ambiguity of individual trials and learn novel words. Their paradigm is known as cross-situational word learning. Although the exact cognitive mechanism involved in this paradigm is still a matter of debate, with some defending a gradual aggregation of information and others a sequential hypothesis testing (for an overview, see Yurovsky & Frank, 2015), there is now evidence suggesting that infants (Smith & Yu, 2008), children (Vlach & DeBrock, 2017), and older adults (Peñaloza et al., 2017) can track word-referent co-occurrences to learn novel words (nouns and verbs; e.g., Fitneva & Christiansen, 2011) in ambiguous contexts (for a recent meta-analysis, (Dal Ben R, Souza DH, Hay JF: Cross-situational word learning: Systematic review and meta-analysis, unpublished)).

Most cross-situational word learning studies use novel words with legal phonotactics (e.g., McGregor et al., 2013; Smith & Yu, 2013), making them plausible labels. However, we do not know whether varying degrees of phonotactic probability impact cross-situational word learning (e.g., Alt et al., 2014). Uncovering any links between phonotactics and cross-situational word learning provides a more comprehensive understanding of how different sources of statistical information may interact to promote or impair word learning (Bohn et al., 2021). This is especially important considering the balance between variability and consistency in word learning in natural environments (Braginsky et al., 2019) and the multimodal nature of statistical language learning (Saffran, 2020; Smith et al., 2018).

For instance, the phonotactic probability of a word might continue to impact its learnability under conditions of ambiguity. Alternatively, the increased task complexity resulting from the inherent ambiguity present in a cross-situational word learning may diminish any potential impact of phonotactics, driving learners to focus on co-occurrences rather than phonological information. To that end, we designed an exploratory study to investigate whether phonotactic knowledge, gathered in pre-experimental experience with natural language, would guide word learning in ambiguous contexts. Critically, all our stimuli had high phonotactic probabilities in Brazilian-Portuguese, the language of our participants. However, some words were slightly more probable than others. We decided to use such subtle differences in phonotactic probabilities for two reasons. First, by having high phonotactic probabilities, all stimuli were good label candidates, but some were better than others. Second, we found that these subtle differences were enough to boost or impair speech segmentation with the same population (Dal Ben et al., 2021) indicating that participants were able to perceive the phonotactic differences between words. Using the same stimuli allowed us to investigate whether or how phonotactic probabilities with known effects in an auditory statistical learning task would be integrated with semantic information to impact cross-situational word learning (e.g., Fitneva et al., 2009; Räsänen & Rasilo, 2015). Our integrative effort is in line with recent discussions on the scope and multimodality of statistical language learning (e.g., Saffran, 2014, 2020; Smith et al., 2014, 2018). Finally, the current study will increase the generalizability of previous findings (e.g., Yu & Smith, 2007; Chen & Yu, 2017) by replicating cross-situational word learning with Brazilian-Portuguese speaking adults.

## Method

### Participants

Thirty adults (*M*_*age*_ = 22.23 years ± 4.5 *SD*, 25 females), all Brazilian-Portuguese native speakers with no reported visual or auditory impairment, participated. They were recruited at the Universidade Federal de São Carlos and received no compensation for their participation (Ethics Committee approval #1.484.847, #3.085.914).

Given the absence of prior research that could inform a power analysis of the impact of phonotactic probabilities on cross-situational word learning, we opted for a sample size that would allow us to capture cross-situational word learning at an above chance level, regardless of the potential effects of phonotactic probabilities. Our sample size was based on Yu and Smith (2007, Experiment 1, 2 × 2 condition), who reported a large effect size of *d* = 4.37 with a sample of 38 adults. A post hoc power analysis (one-sample *t*-test against chance, 0.25, with alpha at 0.05; Faul et al., 2007) estimated that our sample size provided more than 80% of power to detect cross-situational word learning at an above chance level.

### Stimuli

Twelve novel words and twelve novel objects were used. Words came from Dal Ben et al. (2021). To ensure the tight control of phonotactic probabilities, words were created in three steps. First, the algorithm proposed by Vitevitch and Luce’s (2004) was applied to a database of Brazilian-Portuguese biphones (Estivalet & Meunier, 2015) in the following way: Biphones’ log (base 10) phonotactic probabilities were calculated by dividing the sum of the log frequency of each biphone (token) on each word position by the total log frequency of words (token) with biphones in that given position (e.g., log frequency of /mæ/ as the first biphone divided by the total log frequency of all words with at least one biphone). Log transformations were used because they were reported to better correlate with performance in linguistic tasks compared to raw frequency (Vitevitch & Luce, 2004). Second, a search engine was created to find and concatenate biphones. Using this engine, six novel disyllabic words with consonant–vowel structure (CVCV) and with the highest possible phonotactic probability in Brazilian-Portuguese were created (labeled PP+; Table 1). Finally, their biphones were recombined to create other six novel words that had slightly less probable, but still high, phonotactic probabilities (labeled PP−; Table 1). Third, both PP+ and PP− sets were recorded using MBROLA speech synthesizer with the female Brazilian-Portuguese database br4 (Dutoit et al., 1996). Each word lasted for 696 ms, had a mean F0 of 220 Hz, and a mean intensity of 77 dB.

**Table 1:** Phonetic transcription (IPA), Phonotactic Probabilities (PP) of the set with pseudowords with highest possible phonotactic probabilities (PP+) and the set with the lower phonotactic probabilities (PP−)

PP+^**a**^			PP−^**b**^		
	IPA	PP		IPA	PP
dini	[d͡ʒini]	0.0090	nipe	[nipe]	0.0066
deta	[deta]	0.0085	tadi	[tad͡ʒi]	0.0074
pemi	[pemi]	0.0082	mide	[mide]	0.0075
sute	[sute]	0.0084	teba	[teba]	0.0074
viko	[viko]	0.0080	kosu	[kosu]	0.0078
bara	[bara]	0.0090	ravi	[ravi]	0.0073
**Mean**		**0.0085**	**Mean**		**0.0073**

^a^Items with the highest possible phonotactic probabilities (before becoming words) in Brazilian-Portuguese

^b^Items with slightly lower phonotactic probabilities, but that still had relatively high phonotactic probability

Objects were 3D pictures from the NOUN database (Horst & Hout, 2016) with high levels of discriminability (*M* = 90%) and novelty (*M* = 77%). Words and objects were randomly paired. Also, to avoid spurious relations, a counterbalanced version of the pairs was created by switching objects across sets. Participants were randomly assigned to one of these versions. All stimuli are openly available at OSF (https://osf.io/6fqzg/).

### Design

**Fig. 1:**
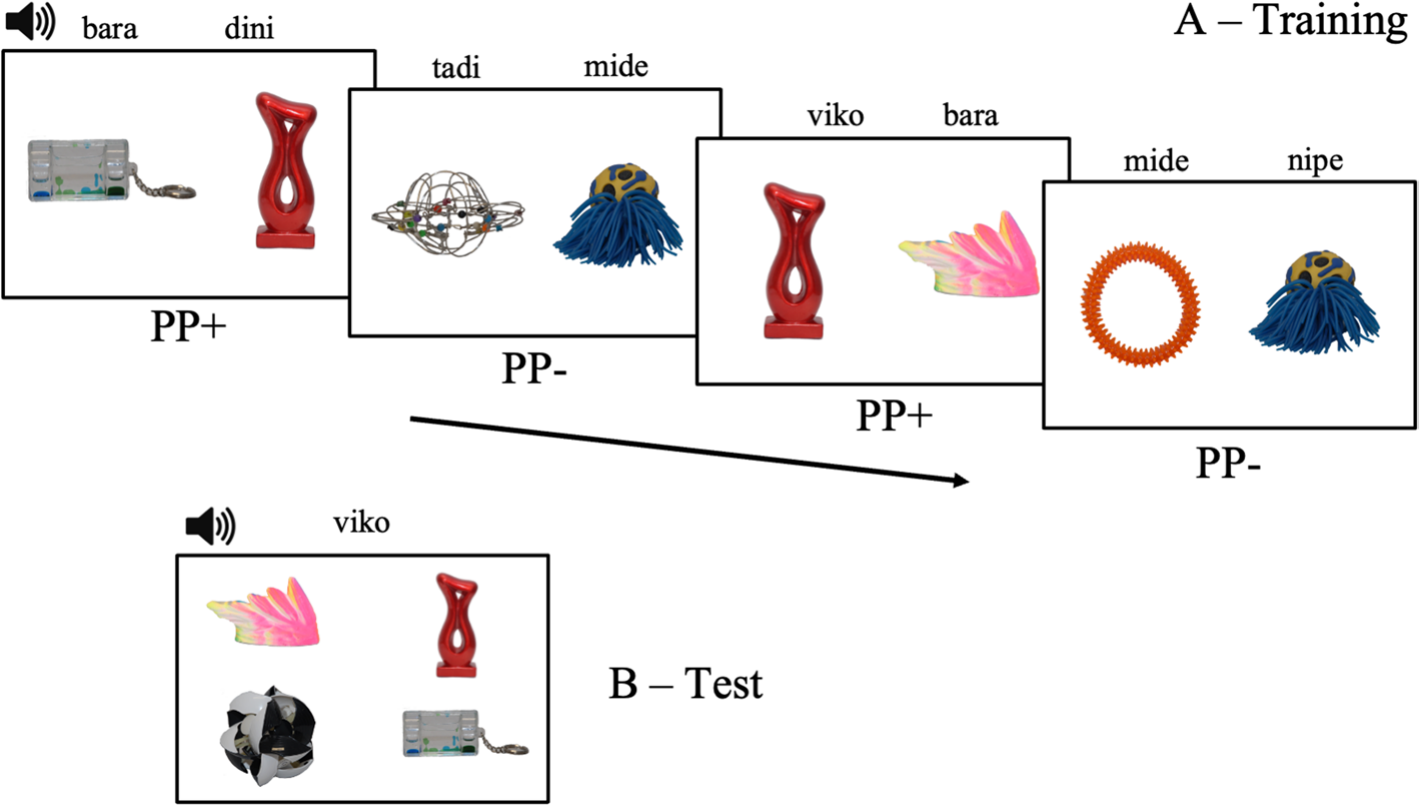
**A** The Training phase with four 2 × 2 trials: two with PP+ pairs and two with PP− pairs. **B** A Test trial (4-alternative forced choice) with PP+ pairs

We used a cross-situational word learning design (Yu & Smith, 2007). The experimental task had two phases: Training and Test. During Training, participants were passively exposed to the 12 word-objects pairs across a series of ambiguous trials (2 × 2; Yu & Smith; Fig. 1). Training trials began with two objects displayed on a screen, side by side. After 950 ms of silence, a word corresponding to one of the objects was played (≈ 696 ms), followed by a silent pause of 700 ms; then another word was played (≈ 696 ms), followed by another silent pause of 950 ms (total duration ≈ 4 s; cf., Yu & Smith). Across trials, there was no reliable correspondence between the position of the objects (left or right) and the order that words were played (first or second). Each of the 12 word-object pairs was presented six times, for a total of 36 trials. Importantly, we used an interleaved presentation of PP+ and PP− word-object pairs. Trials contained words with either higher (PP+) or lower (PP−) phonotactics. Words from different sets never appeared together in the same trial. Thus, for each set, each label was contrasted with the other 5 labels (6 PP+ pairs and 6 PP− pairs).

During the Test phase, each trial began with four objects from the same stimuli set (either PP+ or PP−; never mixed) displayed on each corner of the screen. After 1 s of silence, a word was played (≈ 696 ms), and participants chose the matching object, with no time limit. Each of the 12 word-object pairs were tested twice, for a total of 24 trials (note that we conducted two sets of analyses, one with both trials and another with only the first test trial of each pair; see the Data analysis section). Between each Training and Test trials, a blank screen with a central cross was displayed for 1 s to reorient participants’ gaze to the middle of the screen. Also, between Training and Test, two warm-up trials were conducted to familiarize participants with the structure of the upcoming Test trials. Each warm-up trial displayed four known objects in each corner of the screen (i.e., a house, a duck, a ball, a cat), followed by an audio label of one of the objects (e.g., “House,” in Brazilian-Portuguese). The task was programmed and computer-administered using Psychopy (Peirce et al., 2019).

### Procedure

Participants were seated in a sound-attenuated room in front of a 17″ computer monitor and were fitted with headphones (AKG 240 powered by a Fiio E10K dac/amp). The experimental task began with instrumental music playing at the same volume as the subsequent experimental stimuli (77 dB). Participants were instructed to adjust the volume to a comfortable level. Participants were then instructed that they would: “hear novel words and see novel objects and that their task was to discover word-object relations” (all instructions are available at OSF: https://osf.io/6fqzg/). They were not told that each word corresponded to only one object. Next, the Training phase (as described in the Design section) began and lasted for approximately 3 min. In the subsequent warm-up trials (as described in the Design section), participants were instructed to select the matching object by pressing keys 1, 2, 3, or 4 on a custom keyboard, corresponding to objects in the upper left corner, upper right corner, lower left corner, or lower right corner, respectively. Finally, the Test phase (as described in the Design section) began and lasted for an average of 2 min. To ensure compliance to the instructions, during the entire experiment, participants were monitored by a close-circuit television.

### Data analysis

Our main dependent measure was accuracy (either correct or incorrect selections) during Test trials. However, testing each pair of stimuli twice could have provided participants with additional learning opportunities during Test phase. To account for that, and following literature (e.g., Yu & Smith, 2007), we conducted two sets of analyses: one with both test trials for each pair (24 trials, full dataset) and another with only the first test trial for each pair (12 trials, halved dataset).

For both set of analyses, trials in which reaction times were greater than 3 SDs away from the mean were excluded, as they were most likely the result of participant distraction. Across all participants, a total of 16 trials were excluded from the full dataset and 9 from the halved dataset (either way, 2% of the data). Next, we modeled our binomial (correct or incorrect) and repeated measures (either 24 or 12 trials per participant) using mixed logistic regressions. We used Frequentist and Bayesian approaches (lme4 and brms packages for R; Bates et al., 2015; Bürkner, 2018; R Core Team, 2017). The dependent variable was the selection of the target object during Test (either correct or incorrect). To assess the relationship between phonotactics and above chance word learning, our fixed effects were the chance level (logit of 0.25) and stimuli phonotactics (PP− or PP+). We started with the maximal random structure with pairs (stimuli) as random slopes and participants as random intercepts (Barr et al., 2013). This model failed to converge for the Frequentist approach, but converged for the Bayesian approach, which we report. We then pruned the Frequentist model to include random intercepts for stimuli and participants, this model converged.

It is worth noting that the PP− set was the reference level in the models. Thus, the intercept measures the chances of selecting PP− pairs above chance level (0.25). The odds ratio for selecting PP+ pairs reflect a change in odds from this reference (i.e., PP− above chance level). To arrive at the odds of choosing PP+ pairs, we multiplied the intercept odds by the PP+ odds (Sommet & Morselli, 2017). Finally, given the exploratory nature of our investigation, we do not report *p*-values for our Frequentist analyses (Scheel et al., 2020). Scripts and data are openly available at OSF (https://osf.io/6fqzg/).

## Results and discussion

**Fig. 2:**
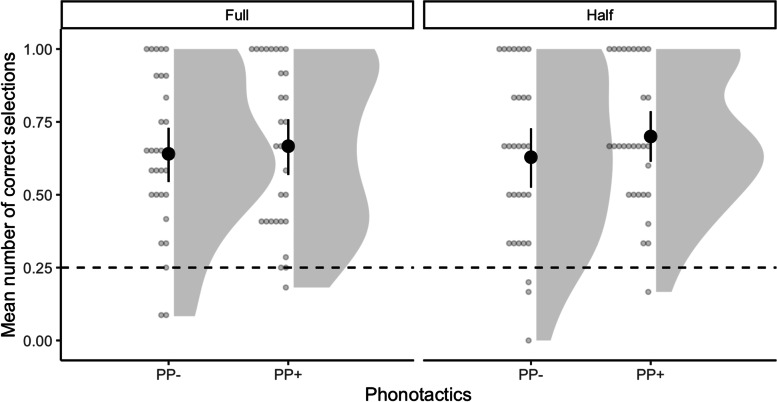
Mean number of correct selections for PP− and PP+ pairs on experiment 1 using the full dataset (24 trials, full) and half of the dataset (12 trials, half). Solid points represent the overall mean; error bars represent 95% CIs (non-parametric bootstrap). Points represent the mean for each participant. The dashed areas depict response distributions. The dashed line represents chance level (0.25)

Results from the full dataset (24 trials) and the halved dataset (12 trials) modeled by Frequentist or Bayesian models were comparable. Participants selected the correct objects above chance level for both PP− and PP+ pairs (Fig. 2). Furthermore, participants were much more likely to choose the correct objects rather than the incorrect ones for both PP− and PP+ pairs (Tables 2 and 3). The odds ratio for choosing PP+ pairs was just slightly higher than the odds ratio for choosing PP. The complete models’ outputs are available at OSF (https://osf.io/6fqzg/).

**Table 2:** Fixed and random effects and specifications for Frequentist models

Model specification (R)	Selection ~ chance level + phonotactics + (1|stimuli) + (1|participant)			
	Full dataset		Half dataset	
Fixed effects				
Predictors	Odds ratio	Confidence interval	Odds ratio	Confidence interval
PP− (intercept)	7.61	3.89–14.90	6.25	3.46–11.29
PP+	1.19	0.83–1.71	1.56	0.95–2.56
Random effects				
σ^2^	3.29		3.29	
*τ*_00 participants_	2.35		1.45	
*τ*_00 stimuli_	0.21		0.09	
ICC	0.44		0.32	
*N*_stimuli_	12		12	
*N*_participants_	30		30	
Observations	704		351	
Marginal *R*^2^/conditional *R*^2^	0.001/0.438		0.010/0.327

**Table 3:** Fixed and random effects and specifications for Bayesian models

Model specification (R)	Selection ~ chance level + phonotactics + (stimuli|participant)			
	Full dataset		Half dataset	
Fixed effects				
Predictors	Odds ratio	Credible interval	Odds ratio	Credible interval
PP− (intercept)	11.93	4.54–35.96	9.42	3.97–28.61
PP+	1.03	0.54–2.01	1.71	0.78–3.87
Random effects				
*σ*^2^	3.29		3.29	
*τ*_00 participants_	5.43		4.10	
ICC	0.62		0.55	
*N*_participants_	30		30	
Observations	704		351	
Marginal *R*^2^/conditional *R*^2^	0.001/0.470		0.007/0.468	

In contrast to evidence suggesting that words with higher phonotactic probabilities are learned faster and more accurately than words with lower phonotactic probabilities (e.g., Gonzalez-Gomez et al., 2013; Estes et al., 2011; Storkel, 2004; Storkel et al., 2013), we found only small differences between PP+ and PP− on our cross-situational word learning task. Indeed, our task was considerably more complex than word learning in unambiguous tasks. On top of having to track word-object co-occurrences across trials to solve referential ambiguity, participants also had to track two independent sets of pairs. Thus, it is unsurprising that participants focused on tracking word-object co-occurrences to solve ambiguities rather than phonological information. Furthermore, the overall accuracy in our task (~ 66%) is comparable to more complex cross-situational word learning studies (e.g., Chen & Yu, 2017; for a recent meta-analysis, (Dal Ben R, Souza DH, Hay JF: Cross-situational word learning: Systematic review and meta-analysis, unpublished)).

These preliminary results prompt a careful examination of the role of phonotactic probabilities in more complex learning environments, with multiple semantic and phonological regularities (Lany & Saffran, 2013; Saffran, 2014; Smith et al., 2014, 2018). Phonotactics might assume different roles depending on environmental complexity. If confirmed by future studies, our exploratory findings might add to the literature pointing to a hierarchical organization of statistical cues as a function of environmental complexity. For instance, in previous research using the same set of stimuli used here, we found that these small differences in phonotactic probabilities could boost or impair speech segmentation based on transitional probabilities (Dal Ben et al., 2021; see also Finn & Hudson Kam, 2008; Mersad & Nazzi, 2011). In the present study, however, phonotactic probabilities might have assumed a secondary role in contrast to word-object co-occurrences. This is in line with previous research suggesting that phonological cues might have limited impact on word learning in ambiguous contexts in comparison to mutual exclusivity based on feedback (Fitneva et al., 2009). On the co-occurrence side, complexity has also been shown to dynamically modulate cross-situational word learning, with learners aggregating information across less complex learning environments and changing to tracking and testing a few label candidates when complexity increases (Yurovsky & Frank, 2015).

As for potential limitations of the current study, both of our stimuli sets (PP+, PP−) had high phonotactic probabilities; thus, words from both sets could be perceived as good label candidates. It is possible that we would have observed a greater influence of phonotactic probability on cross-situational word-learning if their differences had been more salient (e.g., Storkel et al., 2013) or if we had contrasted legal vs. illegal phonotactics (e.g., Estes & Bowen, 2013) or even if PP− and PP+ had been mixed in the same trials (rather than interleaved across trials). Another concern could be that participants may not have perceived the subtle differences in phonotactics between our stimuli. Although previous research investigating speech segmentation with the same set of stimuli suggests participants are indeed sensitive to these subtle differences in phonotactics (Dal Ben et al., 2021), future studies should verify participants’ sensitivity to these phonotactic differences using neurophysiological measures (e.g., EEG), which can reveal implicit perception. Moreover, our sample size was set so it provided enough power to detect an above chance word learning (based on effect sizes reported by Yu & Smith, 2007)—which happened regardless of phonotactic differences between stimuli. When testing the effects of different semantic conditions within a cross-situational word learning task, Chen and Yu (2017) reported a lower effect size than the one we used to inform our power analysis. Mindful of differences in task complexity, future studies could base their power analyses on effect sizes such as those reported by Chen and Yu to ensure enough statistical power to detect small differences between phonotactic probabilities. Finally, as with many studies in the adult psycholinguistic literature, there are at least two potential constraints to our study’s generality (Simons et al., 2017). First, our participants were young college students from a single language background. It is possible that our findings may not generalize to different populations. That said, as the first study to investigate Brazilian-Portuguese speakers in a cross-situational word learning task, our findings do provide evidence for the generality of cross-situational word learning. Second, our study used simplified stimuli (isolated words and highly discriminable objects) in an experimental design. Word learning in everyday life is much more complex, building on several statistical and social cues. Future studies should extend the current research by employing more ecological designs for assessing word learning (e.g., Bergelson et al., 2019; Clerkin et al., 2017).

Mastering a language is a daunting task. Luckily, we can take advantage of environmental regularities to learn a great deal about language. The initial findings we present here move us closer to understanding how regularities in the linguistic input can interact to shape word learning in dynamic environments marked by the constant balance of variance and consistency (Bohn et al., 2021; Braginsky et al., 2019), and ultimately leading to our complex and fascinating linguistic repertoires.

## Data Availability

The datasets analyses scripts, and materials can be found in OSF: https://osf.io/6fqzg/.

## Abbreviations

PP+: Made-up words with highest possible phonotactic probability in Brazilian-Portuguese
PP−: Made-up word with slightly less probable phonotactic probability in Brazilian-Portuguese when compared to PP+ words

## Footnotes

1 Biphone log-based phonotactic probabilities calculated using Vitevitch and Luce (2004) online calculator: fall (/fɔl/), 0.0050; tall (/tɔl/), 0.0044; call (/kɔl/), 0.0060. For details on how these values are calculated, see the Stimuli section.
